# Pre-existing cardiovascular disease and hyperlipidemia and mortality in peritoneal dialysis patients

**DOI:** 10.3389/fendo.2025.1693668

**Published:** 2025-11-11

**Authors:** Panpan Cao, Xiaojiang Zhan, Yueqiang Wen, Xiaoran Feng, Fenfen Peng, Xianfeng Wu, Xiaoyang Wang

**Affiliations:** 1 Department of Nephrology, The First Affiliated Hospital of Zhengzhou University, Zhengzhou, China; 2 Department of Nephrology, the First Affiliated Hospital of Nanchang University, Nanchang, China; 3 Department of Nephrology, the Second Affiliated Hospital of Guangzhou Medical University, Guangzhou, China; 4 Department of Nephrology, Jiujiang No. 1 People’s Hospital, Jiujiang, China; 5 Department of Nephrology, Zhujiang Hospital of Southern Medical University, Guangzhou, China; 6 Department of Nephrology/Clinical Research Center for Chronic Kidney Disease, Affiliated Sixth People’s Hospital, Shanghai Jiao Tong University, Shanghai, China

**Keywords:** chronic kidney disease, peritoneal dialysis, cardiovascular disease, hyperlipidemia, mortality, long-term effect, synergistic effect

## Abstract

**Objectives:**

This study aimed to evaluate the impact of pre-existing cardiovascular disease (CVD) and dyslipidemia on mortality in patients undergoing continuous ambulatory peritoneal dialysis (CAPD).

**Methods:**

This study was conducted as a multicenter retrospective cohort investigation. Eligible patients newly diagnosed with CAPD between January 1, 2005 and December 31, 2018 were enrolled in this study. Missing data were handled using the missForest imputation method. The primary outcome was all-cause mortality. All patients were followed until the cessation of peritoneal dialysis, death, completion of the 8-year follow-up period, or June 30, 2019, whichever occurred first.

**Results:**

Among the 2939 patients, 2132 (72.5%) had no pre-existing CVD or hyperlipidemia, 397 (13.5%) had hyperlipidemia alone, 274 (9.3%) had pre-existing CVD alone, and 136 (4.6%) had pre-existing CVD and hyperlipidemia. The median observational period was 33.6 (IQR 15.6–60.8) months. The number of deaths from all causes were 72 (36.8%), 67 (16.9%), 96 (35.0%), and 306 (14.4%) in the pre-existing CVD plus hyperlipidemia, hyperlipidemia alone, pre-existing CVD alone, and control groups, respectively. After adjusting for confounding factors, patients with pre-existing CVD alone, hyperlipidemia alone, and patients with both conditions had 1.41 (95% CI 1.03 to 1.94), 0.98 (95% CI 0.75 to 1.28) and 1.47 (95% CI 1.16-1.88)-fold greater risk of all-cause mortality, respectively, than patients without pre-existing CVD and hyperlipidemia. Notably, among patients with pre-existing CVD, hyperlipidemic patients had a higher risk of mortality than patients without hyperlipidemia (hazard ratio 0.89, 95% CI 0.61 to 1.31). Among patients without pre-existing CVD, hyperlipidemic patients had a higher risk of mortality than patients without hyperlipidemia (HR 1.10, 95% CI 0.83 to 1.48). There was no interaction effect between the coexistence of pre-existing CVD and hyperlipidemia, pre-existing CVD alone, and hyperlipidemia alone on all-cause mortality (*β* = 0.221, *P* = 0.976).

**Conclusion:**

In patients undergoing CAPD, the coexistence of pre-existing cardiovascular disease and hyperlipidemia is associated with a significantly higher risk of all-cause mortality. This finding suggests that the comorbidity may contribute to worse long-term outcomes and highlights the importance of dyslipidemia management in clinical practice.

## Introduction

In patients with end-stage renal disease (ESRD), cardiovascular disease (CVD) is the major cause of morbidity and mortality, among whom the incidence of coronary artery disease (CAD) exceeds 50% in patients on dialysis (1). Peritoneal dialysis (PD) patients with pre-existing CVD have poorer survival than those without pre-existing CVD (2, 3). In the general population, CAD is mostly attributed to an elevated level of low-density lipoprotein cholesterol (LDL-C), and lipid-lowering therapy is suggested to reduce the risk of CAD (4). However, dyslipidemia in patients with ESRD is characterized by hypertriglyceridemia, and serum total cholesterol (TC) and LDL-C levels are typically within or below normal limits (5). Several studies have shown that lipid-lowering therapy has no beneficial effect on the cardiovascular risk in dialysis patients (6). In addition, ventricular hypertrophy and other nontraditional risk factors, such as anemia, inflammation, chronic kidney disease-mineral bone disorder, and chronic volume overload, also highly contribute to CVD in dialysis patients (1, 7).

The Kidney Disease: Improving Global Outcomes (KDIGO) guidelines suggest that statin treatment is not recommended for chronic dialysis patients (8). However, in a recent study we reported that among PD patients, hyperlipidemia at the beginning of PD was associated with a high risk of long-term mortality (9). Hyperlipidemia is not the main cause of cardiovascular risk in dialysis patients. Thus hyperlipidemia and CVD may have synergistic effects on patient prognosis. The results presented here may provide important guidance for lipid management in PD patients.

## Materials and methods

### Study design and population

A total of 3073 patients with incident continuous ambulatory peritoneal dialysis (CAPD) from five PD centers across three provinces in China were the subjects of our retrospective cohort study (The First Affiliated Hospital of Nanchang University, Nanchang; Zhengzhou University’s First Affiliated Hospital in Zhengzhou; China’s Jiujiang No. 1 People’s Hospital; Southern Medical University’s Zhujiang Hospital in Guangzhou; and Guangzhou Medical University’s Second Affiliated Hospital, Guangzhou), from January 1, 2005, to December 31, 2018. Only patients with no comorbidity records were eliminated to best reflect the real-world situation of the CAPD community. Each research center’s Human Ethics Committee approved the study. The research and clinical trial ethics council at Zhengzhou University’s First Affiliated Hospital disregarded the requirement for informed consent because the data were anonymous.

### Data collection and definitions

Two trained investigators at each center used uniform and standardized data collection tools to aggregate data from medical records on demographics, comorbid conditions, medications, and laboratory values at the beginning of CAPD. Age, sex, body mass index [BMI], systolic blood pressure [SBP], diastolic blood pressure [DBP], 24-hour urine volume, current smoking, and current alcohol consumption were among demographic characteristics. Comorbidities included diabetes mellitus, pre-existing cardiovascular disease, hypertension, and hyperlipidemia. Medications included calcium channel blockers, beta-blockers, diuretics, angiotensin II receptor blockers/angiotensin-converting enzyme inhibitors [ACEI/ARBs], statins, and aspirin; and laboratory variables included hemoglobin, serum albumin, serum uric acid, estimated glomerular filtration rate [eGFR], total cholesterol, triglycerides, high-density lipoprotein cholesterol, low-density lipoprotein cholesterol (LDL-C), and high-sensitivity C-reactive protein [hs-CRP]. SBP > 140 mmHg, DBP > 90 mmHg, or the use of antihypertensive medication were considered indicators of hypertension (10). Coronary heart disease, congestive heart failure, arrhythmia, cerebrovascular illness, or peripheral vascular disease were all considered signs of CVD. Patients with serum levels of total cholesterol ≥ 240 mg/dL, triglycerides ≥ 200 mg/dL, LDL-C > 160 mg/dL, or patients on lipid-lowering medications were considered hyperlipidemic (11). Hyperlipidemia was not used to describe patients with a history of CVD who were taking lipid-lowering drugs to prevent CVD episodes from occurring again. The Chronic Kidney Disease Epidemiology Collaboration equation was used to determine the estimated glomerular filtration rate (12).

### Outcomes and follow-up

All-cause mortality was the primary outcome. Every participant completed CAPD schedules created by dialysis experts in accordance with the patients’ medical conditions and the International Standardized Peritoneal Dialysis Guidelines (13). All patients were followed up until CAPD cessation, death, the end of the 8-year duration, or June 30, 2019. Patients transferring to hemodialysis, receiving a kidney transplant, moving to a different facility, lost to follow-up, continuing to survive after eight years of follow-up, or June 30, 2019, were all censored.

### Statistical analysis

Before the data analysis was conducted, the missing data for the variables were filled in via the missForest method, which accommodates various types of variables. The incidence rate was determined by dividing the number of events by the total valid observational time at risk, expressed as episodes per 1000 years. Variables are reported as the mean ± standard deviation (SD), median (interquartile range, IQR), or number (%). Patients were categorized into four groups: a control group (those without pre-existing CVD or hyperlipidemia), a hyperlipidemia group, a pre-existing CVD group, and a group of patients with both pre-existing CVD and hyperlipidemia. Baseline variables were compared via one-way ANOVA or Kruskal–Wallis tests based on the distribution of the variables (normality assessed with the Shapiro–Wilk test) for quantitative variables and the chi–square test for categorical variables when appropriate. Multinomial logistic regression was performed to assess the relationships between baseline variables and the groups with pre-existing CVD plus hyperlipidemia, pre-existing CVD alone, and hyperlipidemia alone, compared with the control group. Based on previous research evidence and clinical practice experience, and to maintain model simplicity and stability, key pre-specified confounding factors encompass demographic characteristics, major comorbidities, as well as fundamental nutritional and inflammatory status. The following factors were ultimately included in the multinomial logistic regression: age, sex, BMI, systolic blood pressure, current smoking status, current alcohol consumption, diabetes mellitus, hypertension, hemoglobin, serum albumin, serum uric acid, cholesterol, and hs-CRP.Kaplan–Meier curves were generated to examine differences in cumulative mortality among the four groups throughout the observational period. To explore the relationships between these comorbidities and mortality, four Cox proportional hazards regression models were developed and adjusted for various factors: Model 1 was unadjusted; Model 2 included age, sex, BMI, systolic BP, current smoking, current alcohol consumption, diabetes mellitus, and hypertension; Model 3 included medications; and Model 4 included hemoglobin, serum albumin, serum uric acid, cholesterol, and hs-CRP in Model 3. Additionally, the associations were analyzed within subgroups of men, women, those with diabetes mellitus, those without diabetes mellitus, those with hypertension, and those without hypertension. Interactions between sex, diabetes mellitus, and hypertension were tested.The results from the Cox proportional hazards models are reported as hazard ratios (HRs) with 95% confidence intervals (CIs). Statistical analyses were performed via Stata 15.1 software (StataCorp, College Station, TX), with the significance level set at 0.05 for all analyses.

## Results

### Baseline characteristics

Among the 3073 patients, 134 were excluded due to missing records of comorbidities. All variables with less than 5% missing data were imputed before the data analysis, and there were no missing data for the outcomes. Among the 2939 patients, 2132 (72.5%) had no pre-existing CVD or hyperlipidemia, 397 (13.5%) had hyperlipidemia alone, 274 (9.3%) had pre-existing CVD alone, and 136 (4.6%) had pre-existing CVD and hyperlipidemia. The baseline characteristics are shown in **Table 1**. Compared with the control group, the patients with both pre-existing CVD and hyperlipidemia tended to be elderly; were less likely to be male; and had a higher BMI, SBP, and hemoglobin; they were also more likely to have diabetes mellitus or hypertension and take calcium channel blockers, beta-blockers, ACEI/ARBs, aspirin, or statins, but had lower DBP and LDL-C.

**Table 1:** Baseline variables among four groups.

Variables	Control group	Hyperlipidemia	Pre-existing CVD	Pre-existing CVD plus hyperlipidemia	P-value
N	2132	397	274	136	
Age, years	47.0 (37.0-58.0)	52.0 (41.0-61.0)	60.0 (51.0-69.0)	62.0 (51.0-69.0)	<0.001
Male, %	1254 (58.8%)	203 (51.1%)	169 (61.7%)	71 (52.2%)	0.009
Body mass index, kg/m^2^	22.3 (5.4)	23.8 (14.0)	23.0 (3.4)	23.0 (4.8)	0.001
Systolic BP, mmHg	139.1 (25.7)	141.4 (23.9)	142.1 (26.3)	147.2 (25.1)	<0.001
Diastolic BP, mmHg	88.0 (15.9)	87.5 (15.1)	84.2 (15.7)	85.2 (14.3)	<0.001
24-hour urine volume, ml	800.0 (500.0-1200.0)	800.0 (450.0-1200.0)	800.0 (452.5-1200.0)	800.0 (400.0-1300.0)	0.986
Current smoking, (%)	202 (9.5%)	45 (11.3%)	26 (9.5%)	21 (15.4%)	0.112
Current alcohol consumption, (%)	75 (3.5%)	20 (5.0%)	7 (2.6%)	6 (4.4%)	0.332
Diabetes mellitus, (%)	266 (12.5%)	106 (26.7%)	105 (38.3%)	72 (52.9%)	<0.001
Hypertension, (%)	1291 (60.6%)	270 (68.0%)	236 (86.1%)	118 (86.8%)	<0.001
Calcium channel blockers, (%)	1524 (71.5%)	337 (84.9%)	213 (77.7%)	127 (93.4%)	<0.001
Beta blockers, (%)	814 (38.2%)	208 (52.4%)	109 (39.8%)	82 (60.3%)	<0.001
Diuretics, (%)	143 (6.7%)	8 (2.0%)	38 (13.9%)	11 (8.1%)	<0.001
ACEI/ARBs, (%)	645 (30.3%)	181 (45.6%)	115 (42.0%)	71 (52.2%)	<0.001
Aspirin, (%)	128 (6.0%)	28 (7.1%)	55 (20.1%)	33 (24.3%)	<0.001
Statins, (%)	206 (9.7%)	92 (23.2%)	63 (23.0%)	55 (40.4%)	<0.001
Hemoglobin, g/dL	9.0 (2.8)	9.8 (2.6)	9.4 (2.7)	10.6 (2.8)	<0.001
Serum albumin, g/dL	3.5 (0.6)	3.4 (0.5)	3.5 (0.6)	3.5 (0.6)	0.345
Serum uric acid, mg/dL	7.0 (2.4)	6.9 (2.3)	6.7 (2.1)	6.8 (2.4)	0.302
eGFR, mL/min/1.73 m^2^	6.5 (4.7-8.3)	6.4 (4.7-8.3)	5.9 (4.6-8.4)	6.5 (4.9-8.3)	0.555
Total cholesterol, mg/dL	149.3 (116.3-181.7)	152.7 (119.9-184.7)	156.0 (125.6-185.6)	157.4 (129.0-186.9)	0.053
Triglyceride, mg/dL	94.8 (57.6-153.3)	87.7 (52.3-147.8)	95.7 (46.7-157.8)	113.0 (68.0-164.5)	0.288
HDL-C, mg/dL	39.6 (31.3-49.9)	39.4 (31.4-49.5)	38.1 (29.8-49.9)	41.6 (32.5-47.6)	0.737
LDL-C, mg/dL	80.5 (48.3-116.1)	85.5 (56.8-122.2)	83.9 (41.8-118.6)	74.2 (19.4-116.2)	0.017
hs-CRP, mg/L	4.3 (1.9-13.6)	4.4 (1.9-14.2)	4.5 (1.9-18.9)	5.0 (1.9-18.6)	0.703

CVD, cardiovascular disease; BP, blood pressure; ACEI/ARBs, angiotensin II receptor blockers/angiotensin-converting enzyme inhibitors; eGFR, estimated glomerular filtration rate; HDL-C, High density lipoprotein cholesterol; LDL-C, Low density lipoprotein cholesterol; hs-CRP, high-sensitivity C-reactive protein.

### Associations between baseline variables and pre-existing CVD plus hyperlipidemia, preexisting CVD alone, or hyperlipidemia alone

We analyzed baseline variables of patients associated with the coexistence of pre-existing CVD plus hyperlipidemia, pre-existing CVD alone, or hyperlipidemia alone versus patients without the two conditions via multinomial logistic regression (**Table 2**). After adjusting for confounding factors, diabetes mellitus and hypertension were associated with a greater risk of patients having pre-existing CVD coexisting with hyperlipidemia, pre-existing CVD alone, or hyperlipidemia alone than the risk in patients without these two conditions. Older age, female sex, current smoking status, higher systolic BP, and higher hemoglobin levels were associated with a greater risk of pre-existing CVD coexisting with hyperlipidemia. Female sex, a higher body mass index, and higher hemoglobin levels were associated with a greater risk of hyperlipidemia. Older age and higher hemoglobin levels were associated with a greater risk of pre-existing CVD. Notably, diabetes mellitus was associated with a 4.13 (95% CI 2.75 to 6.19)-fold risk of pre-existing CVD coexisting with hyperlipidemia, followed by hypertension, with a 2.27 odds ratio (95% CI 1.33 to 3.87).

**Table 2:** Associations between variables at baseline and co-existence of pre-existing CVD and hyperlipidemia, pre-existing CVD, and hyperlipidemia.

Variables	Control group	Hyperlipidemia		Pre-existing CVD		Pre-existing CVD plus hyperlipidemia	
		OR	95% CI	OR	95% CI	OR	95% CI
Age, per increase 10 years	1.0 (ref.)	1.08	0.99 to 1.17	1.62	1.47 to 1.79	1.55	1.35 to 1.79
Female, male as a reference	1.0 (ref.)	1.56	1.24 to 1.97	0.85	0.64 to 1.13	1.57	1.06 to 2.33
Body mass index, per increase 1 kg/m^2^	1.0 (ref.)	1.02	1.01 to 1.03	1.01	0.99 to 1.03	1.00	0.95 to 1.04
Systolic BP, per increase 10 mmHg	1.0 (ref.)	1.02	0.97 to 1.07	1.01	0.95 to 1.08	1.10	1.01 to 1.19
Current smoking, yes/no	1.0 (ref.)	1.23	0.81 to 1.88	1.06	0.69 to 1.62	2.18	1.32 to 3.59
Diabetes mellitus, yes/no	1.0 (ref.)	2.21	1.66 to 2.93	2.14	1.58 to 2.89	4.13	2.75 to 6.19
hypertension, yea/no	1.0 (ref.)	1.14	0.90 to 1.46	2.80	1.93 to 4.06	2.27	1.33 to 3.87
Hemoglobin, per increase 1 g/dL	1.0 (ref.)	1.10	1.06 to 1.14	1.03	0.98 to 1.07	1.16	1.10 to 1.23

Variables adjusted in the multinomial logistic regression model included: age, sex, body mass index, systolic BP, diastolic BP, current smoking, current alcohol consumption, 24-hour urine volume, diabetes mellitus, hypertension, hemoglobin, serum albumin, serum uric acid, eGFR, cholesterol, triglyceride, high density lipoprotein, low density lipoprotein, and hs-CRP.

CVD, cardiovascular disease; BP, blood pressure; eGFR, estimated glomerular filtration rate; hs-CRP, high-sensitivity C-reactive protein; OR, odds ratio; CI, confidence interval.

### Observational period and mortality

The median observational period was 33.6 (IQR 15.6–60.8) months. During this period, 541 (18.4%) patients died, 370 (12.6%) were transferred to hemodialysis, 156 (5.3%) received renal transplants, 25 (0.8%) were transferred to other dialysis centers, and 103 (3.5%) were lost to follow-up. The number of deaths from all causes were 72 (36.8%), 67 (16.9%), 96 (35.0%), and 306 (14.4%) in the pre-existing CVD plus hyperlipidemia, hyperlipidemia alone, pre-existing CVD alone, and control groups, respectively.The incidence rates of all-cause mortality were 195.6, 52.4, 105.1, and 40.5 per 1000 patient-years among the pre-existing CVD plus hyperlipidemia, hyperlipidemia alone, pre-existing CVD alone, and control groups, respectively (**Table 3**).

**Table 3:** All-cause mortality incidence among four groups. .

Groups	Number of events (%)	Time at risk (years)	Incidence (per 1000 patient-years)
Control group	306 (14.4%)	7562.8	40.5
Hyperlipidemia	67 (16.9%)	1277.6	52.4
Pre-existing CVD	96 (35.0%)	913.6	105.1
Pre-existing CVD plus hyperlipidemia	72 (36.8%)	368.2	195.6

Incidence was calculated as number of events divided by total valid observational time at risk, scaled to episodes per 1000 years.

CVD, cardiovascular disease; CI, confidence interval.

### Comorbidities and mortality

**Figure 1:**
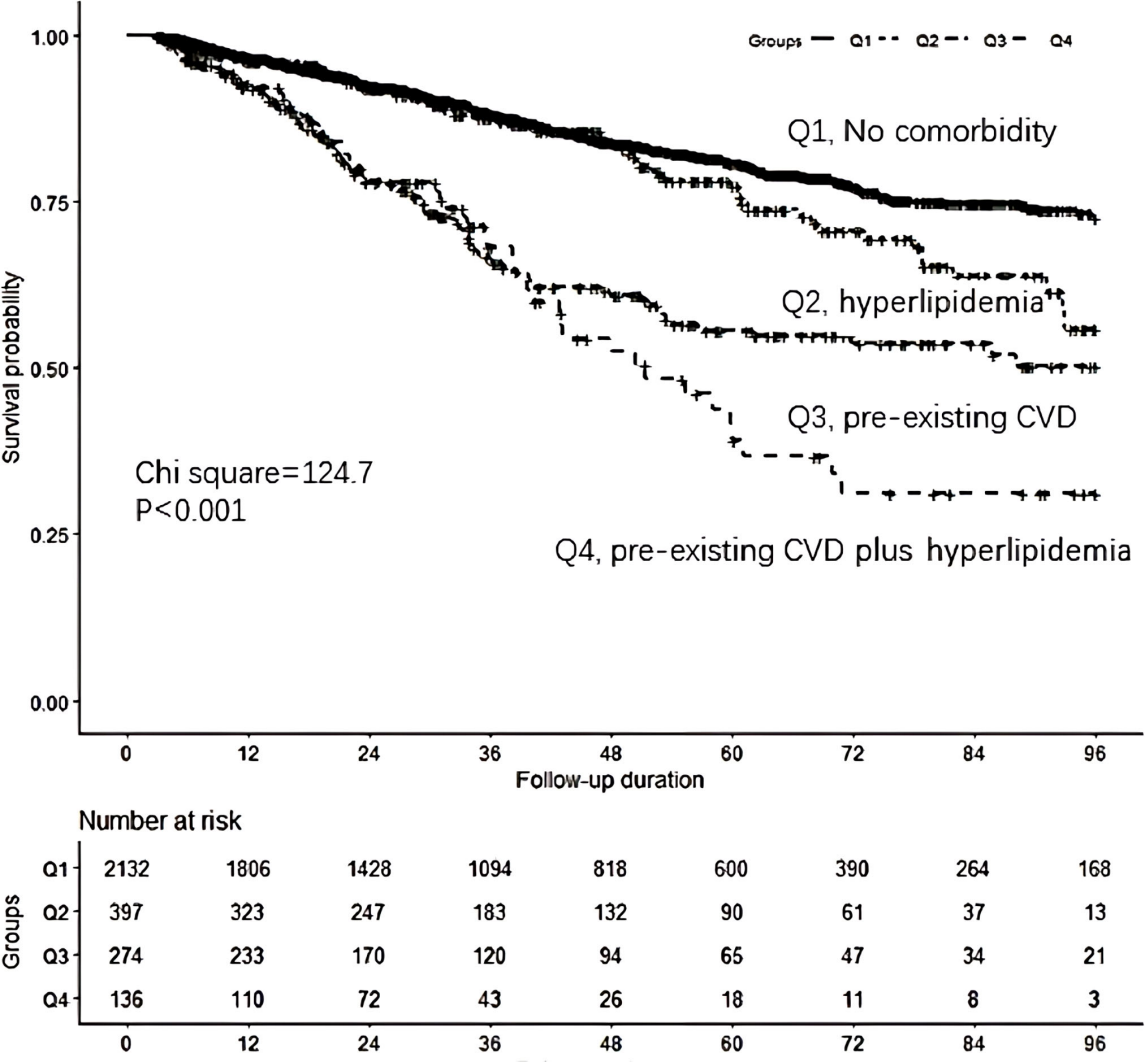
Comparison of survival curves according to the four groups.Q1: the control group (those without pre-existing CVD or hyperlipidemia), Q2:hyperlipidemia group, Q3: pre-existing CVD group, Q4:pre-existing CVD I plus hyperlipidemia group.Survival analysis found that the pre-existing CVD plus hyperlipidemia group had greater cumulative all-cause mortality (P <0.001)compared to the control group.

Survival analysis revealed that the pre-existing CVD plus hyperlipidemia group had greater cumulative all-cause mortality (*P* < 0.001) than the control group (**Figure 1**). The associations between comorbidities and mortality were evaluated via different Cox proportional hazards regression models (**Table 4**). Compared with the control group, the pre-existing CVD plus hyperlipidemia, hyperlipidemia alone, and pre-existing CVD alone groups had 1.47 (95% CI 1.16 to 1.88), 0.98 (95% CI 0.75 to 1.28), and 1.41 (95% CI 1.03 to 1.94)-fold greater risks of all-cause mortality, respectively, than the control group in Model 4. Similar trends were observed among the male, female, diabetes mellitus, nondiabetes mellitus, hypertension, and nonhypertension subgroups (data not shown). There was no interaction effect of the coexistence of pre-existing CVD and hyperlipidemia, pre-existing CVD alone, or hyperlipidemia alone on all-cause mortality (*β* = 0.221, *P* = 0.976). The P values for interactions were > 0.05 for the subgroups by sex (*β* = 0.128, *P* = 0.825), diabetes mellitus (*β* = 0.221, *P* = 0.764), and hypertension (*β* = 0.218, *P* = 0.758). The P values for interactions were > 0.05 for all subgroups, suggesting that the increased risk of all-cause mortality associated with interesting comorbidities was evident regardless of these subgroup variables.

**Table 4:** Hazards ratios for all-cause mortality among different groups using Cox regression.

Groups	Model 1		Model 2		Model 3		Model 4	
	HR	95%CI	HR	95%CI	HR	95%CI	HR	95%CI
Control group	1.0 (ref.)							
Hyperlipidemia	1.31	1.01 to 1.70	0.99	0.75 to 1.29	0.99	0.75 to 1.29	0.98	0.75 to 1.28
Pre-existing CVD	2.63	2.09 to 3.31	1.42	1.03 to 1.95	1.42	1.03 to 1.96	1.41	1.03 to 1.94
Pre-existing CVD plus hyperlipidemia	3.46	2.57 to 4.67	1.45	1.14 to 1.85	1.45	1.14 to 1.85	1.47	1.16 to 1.88

Model 1, unadjusted; model 2, model 1 plus age, sex, body mass index, systolic BP, diastolic BP, current smoking, current alcohol consumption, 24-hour urine volume, diabetes mellitus, and hypertension; model 3, model 2 plus medications; model 4, model 3 plus hemoglobin, serum albumin, serum uric acid, eGFR, cholesterol, triglyceride, high density lipoprotein, low density lipoprotein, and hs-CRP.

CVD, cardiovascular disease; BP, blood pressure; eGFR, estimated glomerular filtration rate; hs-CRP, high-sensitivity C-reactive protein; HR, hazard ratio; CI, confidence interval.

Compared with patients with hyperlipidemia (reference), those with pre-existing CVD had a greater risk of all-cause mortality (HR 1.59, 95% CI 1.16 to 2.20; **Table 5**). In hyperlipidemic patients, pre-existing CVD was associated with a 1.86-fold greater risk of all-cause mortality (95% CI 1.22 to 2.81) than nonpreexisting CVD was. During the follow-up period, hyperlipidemia was not associated with a greater risk of all-cause mortality than nonhyperlipidemia was (HR 0.89, 95% CI 0.61 to 1.31) in patients with pre-existing CVD alone (**Table 6**). However, from the 42-month follow-up onward, hyperlipidemia was associated with a greater risk of all-cause mortality compared with non-hyperlipidemia (HR 8.91, 95% CI 4.56 to 18.80). Additionally, from the 60-month follow-up onward, hyperlipidemia was also associated with a greater risk of all-cause mortality compared with non-hyperlipidemia (HR 5.58, 95% CI 3.62 to 8.60) in those without pre-existing CVD alone.

**Table 5:** Hazards ratios for all-cause mortality using Cox regression.

Groups	Model 1		Model 2		Model 3		Model 4	
	HR	95%CI	HR	95%CI	HR	95%CI	HR	95%CI
Hyperlipidemia	1.0 (ref.)							
Pre-existing CVD	2.00	1.47 to 2.74	1.58	1.14 to 2.18	1.58	1.14 to 2.66	1.59	1.16 to 2.20
Hyperlipidemia patients								
Pre-existing CVD (yes/no)	2.78	1.92 to 4.02	1.53	1.03 to 2.27	1.68	1.12 to 2.50	1.86	1.22 to 2.81

Model 1, unadjusted; model 2, model 1 plus age, sex, body mass index, systolic BP, diastolic BP, current smoking, current alcohol consumption, 24-hour urine volume, diabetes mellitus, and hypertension; model 3, model 2 plus medications; model 4, model 3 plus hemoglobin, serum albumin, serum uric acid, eGFR, cholesterol, triglyceride, high density lipoprotein, low density lipoprotein, and hs-CRP.

CVD, cardiovascular disease; BP, blood pressure; eGFR, estimated glomerular filtration rate; hs-CRP, high-sensitivity C-reactive protein; HR, hazard ratio; CI, confidence interval.

**Table 6:** Associations between hyperlipidemia and all-cause mortality in patients with or without pre-existing CVD.

Groups	Model 1		Model 2		Model 3		Model 4	
	HR	95%CI	HR	95%CI	HR	95%CI	HR	95%CI
Pre-existing CVD patients								
The follow-up period								
Hyperlipidemia (yes/no)	1.27	0.90 to 1.79	0.98	0.68 to 1.41	0.90	0.62 to 1.31	0.89	0.61 to 1.31
From 42-month follow up onwards								
Hyperlipidemia (yes/no)	8.39	4.49 to 15.68	6.44	3.32 to 12.48	6.03	3.08 to 11.82	8.91	4.56 to 18.80
Non-pre-existing CVD patients								
The follow-up period								
Hyperlipidemia (yes/no)	1.31	1.01 to 1.71	0.98	0.75 to 1.29	1.06	0.80 to 1.40	1.10	0.83 to 1.48
From 60-month follow up onwards								
Hyperlipidemia (yes/no)	7.67	5.12 to 11.49	5.21	3.39 to 8.01	5.21	3.39 to 8.01	5.58	3.62 to 8.60

Model 1, unadjusted; model 2, model 1 plus age, sex, body mass index, systolic BP, diastolic BP, current smoking, current alcohol consumption, 24-hour urine volume, diabetes mellitus, and hypertension; model 3, model 2 plus medications; model 4, model 3 plus hemoglobin, serum albumin, serum uric acid, eGFR, cholesterol, triglyceride, high density lipoprotein, low density lipoprotein, and hs-CRP.

CVD, cardiovascular disease; BP, blood pressure; eGFR, estimated glomerular filtration rate; hs-CRP, high-sensitivity C-reactive protein; HR, hazard ratio; CI, confidence interval.

## Discussion

CVD is highly prevalent in dialysis patients and accounts for up to 40% of deaths in those patients (14). Previous lipid-lowering intervention studies had relatively short follow-up durations, which is not enough to observe the long-term prognosis (6). Therefore, the adverse effects of dyslipidemia in PD dialysis patients may be underestimated. we conducted this observational study to assess whether there was an additive effect of the coexistence of pre-existing CVD and hyperlipidemia on mortality in PD patients.

This retrospective study analyzed 2,939 peritoneal dialysis patients over 8 years of follow-up. The results demonstrated that compared with patients without pre-existing CVD and hyperlipidemia, patients with both pre-existing CVD and hyperlipidemia had the highest risk of all-cause mortality (HR 1.47, 95% CI 1.16 to 1.88), whereas patients with pre-existing CVD alone and hyperlipidemia alone had 1.41 (95% CI 1.03 to 1.94) and 0.98 (95% CI 0.75 to 1.28) times greater risks of all-cause mortality. It seems that hyperlipidemia has no effect on PD mortality, but in the hyperlipidemia cohort, patients with pre-existing CVD had a 1.86 (95% CI 1.22 to 1.81)-fold greater risk of all-cause mortality than patients without pre-existing CVD. Among pre-existing CVD cohort patients, patients with hyperlipidemia had an 8.91 (95% CI 4.56 to 18.80)-fold greater risk of all-cause mortality than patients without hyperlipidemia did from the 42-month follow-up onward. Our previous study revealed that, from the 48-month follow-up onward, hyperlipidemia was associated with a 2.26 (95% CI 1.49 to 3.43)-fold greater risk of all-cause mortality than nonhyperlipidemia was (9). Another study indicated that from the 48-month follow-up onward, hyperlipidemic patients had a 3.60 (95% CI 1.62 to 8.01)-fold greater risk of all-cause mortality than nonhyperlipidemic PD patients with diabetes (15). Based on the statistical observations from the above data, it is suggested that among CAPD patients, those with pre-existing CVD and hyperlipidemia were at the highest risk of 8-year all-cause mortality, followed by pre-existing CVD alone patients and hyperlipidemia alone patients. Hyperlipidemia has an adverse effect on long-term survival among CAPD patients, especially patients with pre-existing CVD. However, reports on this synergistic effect remain limited, and its exact causal mechanism remains unclear. Therefore, future validation through more prospective studies is warranted.

Given the complex nature of dyslipidemia in peritoneal dialysis patients, although we included those with high lipid profiles according to the 2016 Chinese Guidelines for Managing Hyperlipidemia in Adults, 29434322, rather than limiting to hypertriglyceridemia alone, this approach may still lead to underestimation of dyslipidemia prevalence (5). Some previous studies have suggested that due to high glucose absorption from the peritoneal dialysate and protein loss from the peritoneum, the lipid profile of PD patients is more atherogenic than that of hemodialysis patients (16). While Gokhan Aydin et al. found no significant differences in atherogenic lipid parameters based on glucose absorption levels or dialysis modality (17). The discussion on this topic remains inconclusive. Furthermore, studies have demonstrated that prolonged peritoneal dialysis duration increases the incidence of new-onset hyperglycemia and obesity (18), and these unique lipid metabolism influencers are not observed in hemodialysis. However, a meta-analysis of Chinese diabetic nephropathy patients revealed that compared to HD patients, PD patients demonstrated higher cholesterol and triglyceride levels yet significantly lower cardiovascular events. The analysis suggested that patients with higher cardiovascular risk may be more suitable for PD than HD (19). This indicates that switching from PD to HD may not be an effective solution to address the fundamental issue.

Statins, 3-hydroxy-3-methylglutaryl coenzyme A (HMG-CoA) reductase inhibitors, are known as lipid-lowering agents and have anti-inflammatory effects (20). Statin therapy is associated with lower CRP levels in both hemodialysis and PD patients (21, 22). Statin therapy may reduce cardiovascular event mortality and morbidity in dialysis patients. However, previous studies have shown that statin therapy has no beneficial effect on the cardiovascular risk in dialysis patients (6). This is related to the fact that statin therapy targets a single pathway while the lipid metabolism mechanisms in dialysis patients are complex (23, 24). In PD patients, exposure to PD catheters, bioincompatible dialysate, and PD-related peritonitis, among other factors, may accelerating the development of CVD (25). The pathogenesis involves multiple mechanisms: such as oxidative stress and inflammation, hyperactive renin–angiotensin–aldosterone system, maladaptive Wnt/β-catenin signalling pathway and profibrotic TGF–β1/Smad signalling pathway (24, 26, 27), as well as other pathogeneses, such as dysbiosis of the gut microbiota and dysregulation of noncoding RNAs (28, 29). However, the specific mechanisms remain unclear. For PD patients, an ideal lipid-lowering drug may need to demonstrate efficacy in improving nutritional status, modulating inflammatory states, and reducing lipid levels. This requires further research to identify newer and more precise therapeutic targets. Currently, no single medication can achieve all these goals (24). A comprehensive approach combining lifestyle modifications, nutritional supplementation, anti-inflammatory medications, and improved dialysis therapies may be necessary to effectively manage dyslipidemia (24, 30).

The strengths of this study included a large sample size, a population from five dialysis centers, and a detailed evaluation and adjustment for all-cause risk factors for real-world data. However, this study has several limitations. First, the relatively low baseline proportions of patients with both pre-existing CVD and hyperlipidemia and those with hyperlipidemia alone may limit the statistical power in subgroup analyses. Second, as all patients were from China, and considering the impact of dietary differences across countries on lipid levels, the findings may lack generalizability to other ethnic populations. Third, all-cause mortality served as the primary outcome due to unavailable cause-specific data, limiting the interpretation of cardiovascular mortality. Future prospective interventional studies are needed to obtain more reliable evidence.

## Conclusions

Compared with patients without pre-existing CVD and hyperlipidemia, CAPD patients with both pre-existing CVD and hyperlipidemia were at higher risk of 8-year all-cause mortality. Hyperlipidemia has long-term adverse effects on PD patients, especially patients with pre-existing CVD. Therefore, lipid-lowering therapy may be suggested for PD patients with both hyperlipidemia and pre-existing CVD. Future prospective interventional studies are needed to obtain more reliable evidence.

## Data Availability

The raw data supporting the conclusions of this article will be made available by the authors, without undue reservation.
